# Transcriptional enhancers and their communication with gene promoters

**DOI:** 10.1007/s00018-021-03903-w

**Published:** 2021-08-19

**Authors:** Helen Ray-Jones, Mikhail Spivakov

**Affiliations:** 1 MRC London Institute of Medical Sciences, London, W12 0NN UK; 2 Institute of Clinical Sciences, Faculty of Medicine, Imperial College, London, W12 0NN UK

**Keywords:** Transcriptional enhancers, Enhancer–promoter interactions, Chromosomal conformation, Gene regulation

## Abstract

Transcriptional enhancers play a key role in the initiation and maintenance of gene expression programmes, particularly in metazoa. How these elements control their target genes in the right place and time is one of the most pertinent questions in functional genomics, with wide implications for most areas of biology. Here, we synthesise classic and recent evidence on the regulatory logic of enhancers, including the principles of enhancer organisation, factors that facilitate and delimit enhancer–promoter communication, and the joint effects of multiple enhancers. We show how modern approaches building on classic insights have begun to unravel the complexity of enhancer–promoter relationships, paving the way towards a quantitative understanding of gene control.

## Introduction

In complex organisms, the vast majority of genes are not controlled by promoters alone, but additionally receive input from one or more non-coding DNA *cis*-regulatory elements, the best-characterised of which are enhancers. The first transcriptional enhancers were identified about 40 years ago, and their critical role in development has been clear for at least two decades. Advances in recent years have led to the identification of millions of enhancers active in an ever-expanding array of cell types. Meanwhile, population genetics studies have revealed the enrichment of these elements for genetic variants associated with common diseases. Most recently, targeted perturbation and imaging techniques have taken functional analyses of enhancers to a new level of scale and resolution. As the quest for a mechanistic understanding of enhancers continues, the function of these elements emerges as a complex phenomenon that integrates multiple levels of nuclear organisation, from primary DNA sequence and sequence-specific transcription factors to higher-order chromatin architecture through chromatin remodelling complexes, chromosomal loops and potentially phase-separated condensates. In this review, we synthesise classic and recent evidence on the organisation and function of enhancers, focusing in particular on the principles governing their communication with target gene promoters.

## Organisation and function of enhancers *in cis*

### Classic definition of enhancers

Enhancers were first described around 20 years after the discovery of the gene promoter [1–7]. In 1980, the first evidence for enhancers arose when short DNA sequences were discovered within the simian virus 40 (SV40) [2] and the sea urchin genome [3] that were remote from a gene promoter, yet seemed to stimulate gene expression by an unknown mechanism. Subsequent landmark experiments by the groups of Walter Schaffner [4] and Pierre Chambon [5] confirmed that a 72-bp repeat sequence element in SV40 [8, 9] was an “enhancing” sequence, capable of vastly upregulating gene expression from a plasmid upon transfection into mammalian cells. Moreover, this enhancer could activate rabbit or human *β-globin* genes from varying distances from their promoters, regardless of its orientation [4, 5]. At around the same time, similar observations were made for an unrelated enhancer sequence within the polyoma virus [10]. We now know that the ability to function remotely and independently of orientation are classic features of enhancers. The SV40 discovery was useful for identifying further enhancers via the “enhancer trap” method: an SV40 genome lacking its known enhancer was combined with fragments of other viruses and transfected into mammalian cells. DNA combinations with the ability to replicate must have gained an enhancer from the new fragment [11, 12].

Enhancers were soon discovered in the genomes of other model organisms in a range of complexities from yeast [13] to *Drosophila* [14] and mouse [15–17]; enhancer-like sequences were also detected in bacteria [18–22]. In *Drosophila*, newly discovered enhancers orchestrated the regulation of developmental genes that were crucial for the correct formation of the embryo [23, 24]. It soon became clear that enhancers also played vital roles in humans, with non-coding enhancer deletions starting to be linked to severe disease phenotypes such as beta thalassaemia and polydactyly [25–28].

### Enhancers as transcription factor recruitment units

**Fig. 1:**
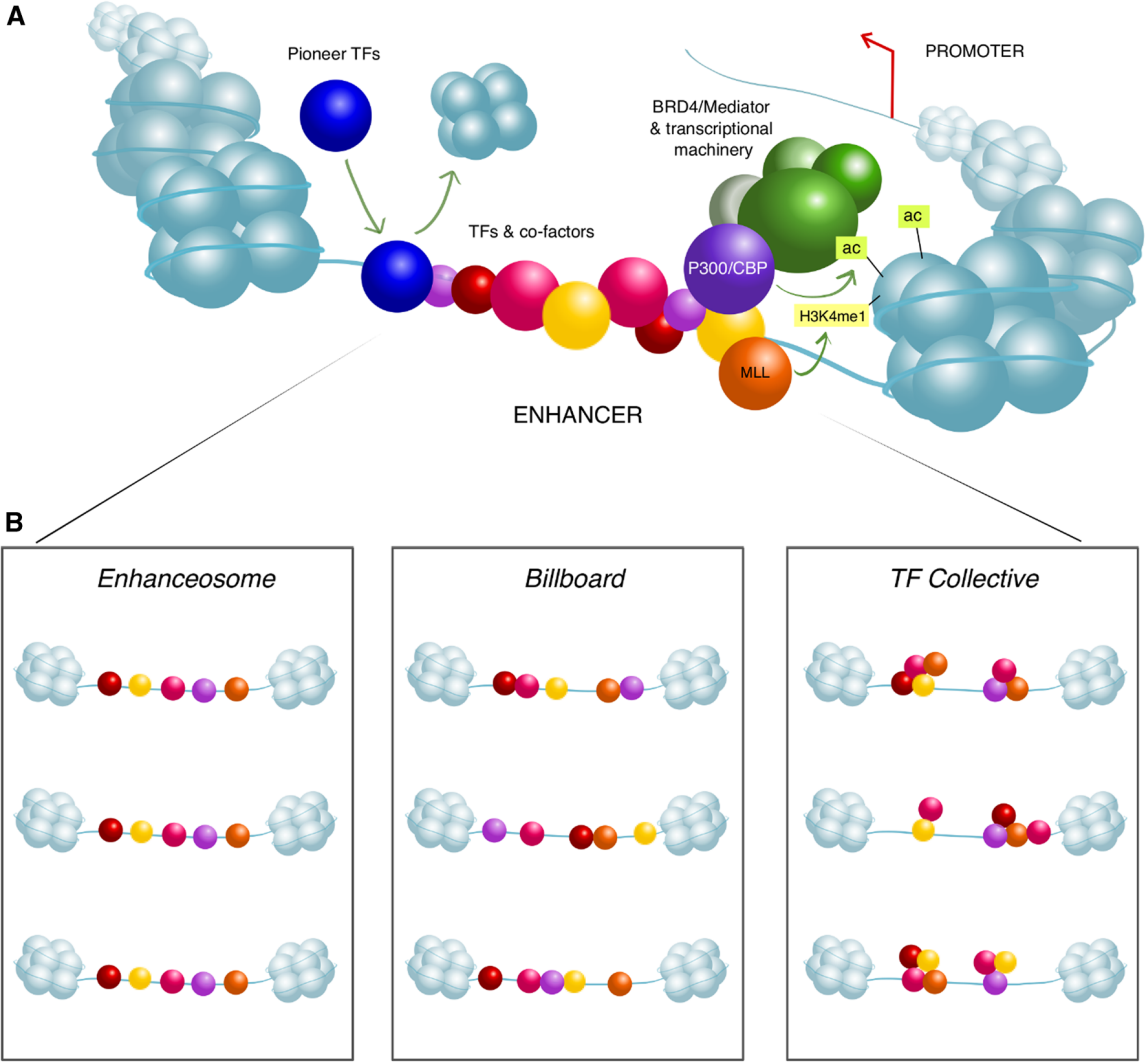
Organisation of active enhancers *in cis*. **A** Enhancers become activated by pioneer factors that increase chromatin accessibility, perhaps through histone displacement, allowing for binding of cell-specific TFs and cofactors. Cofactors such as P300 and MLL perform chromatin remodelling via acetylation or methylation of histone tails. TFs and cofactors recruit BRD4/Mediator and further transcriptional machinery to begin active transcription at the enhancer and the distal gene promoter. **B** Models of enhancer grammar include the rigid Enhanceosome model [88], in which TFs bind in a particular order and spacing; the Billboard model, in which TFs bind in a very flexible arrangement [90], and the TF Collective model, in which a full set of particular TFs is required for enhancer activation, but not all TFs must bind directly to the DNA and instead are recruited via protein–protein interactions [91]

At the DNA level, enhancers represent clusters of binding sites for sequence-specific transcription factors (TFs) (Fig. 1A) (reviewed in [29, 30]). However, the ability of enhancers to recruit their cognate TFs may be constrained by inaccessible (“closed”) chromatin conformation, whereby enhancer regions are tightly packaged in nucleosomes. A subset of TFs known as pioneer factors are capable of overcoming this constraint through chromatin remodelling (reviewed in [31, 32]). Pioneer factors play particularly important roles in priming enhancers in order to program gene expression patterns during early development. In *Drosophila*, the Zinc Finger Early Drosophila Activator (Zelda) is critical for lowering the nucleosome barrier at enhancers for genes driving zygotic gene activation [33–35]. In mice and humans, the pioneer factors OCT4 and SOX2 form a core part of the regulatory network controlling stem cell pluripotency [36]. The mechanisms of pioneer factor action are still being established [37], but at least some of them (such as FOXA, OCT4 and SOX2/SOX11) have been shown to initiate chromatin opening by direct displacement of histones [38–41], whilst many others recruit chromatin remodelers [42–50]. Often, pioneer factors must recruit additional non-pioneer sequence-specific TFs for efficient enhancer activation [30, 31]. For instance, Zelda promotes the formation of local TF hubs involving important transcription factors required for developmental patterning, such as Bicoid and Dorsal [51–54]. Synthetic enhancers recruiting multiple heterotypic TFs showed stronger transcriptional effects compared with those recruiting high amounts of a single TF, providing a functional rationale for combinatorial TF recruitment to natural enhancers [55]*.*

Mechanistically, a key function of sequence-specific TFs is the recruitment of “workhorse” cofactors that facilitate enzymatic chromatin remodelling, histone modification and act as scaffolds for recruitment of additional factors. These cofactors include, but are not limited to, SWI/SNF and FACT chromatin remodelling complexes, P300 and CBP chromatin activators, Bromodomain-containing (BRD) proteins and the Mediator complex [56, 57]. The SWI/SNF complex is involved in regulating occupancy and spacing of nucleosomes at promoters and enhancers [58], while the most established role of FACT is in facilitating transcriptional elongation [57]. SWI/SNF recruits the chromatin activators P300 and CBP, which mediate the acetylation of histone H3 lysine 27 through their intrinsic histone acetyltransferase (HAT) activity [59–62]. This P300/CBP-induced acetylation has recently been implicated in the release of “paused” RNAP at both enhancers and promoters to promote active transcription [63]. P300 and CBP also serve as scaffolds that connect RNA polymerase II (RNAP) and RNAP-associated “general transcription factors” to the chromatin [64, 65]. At active enhancers, the BRD4 protein co-localises with the Mediator complex, which in turn assembles the pre-initiation complex (PIC) and RNAP to initiate transcription [66–68].

Recent experiments degrading Mediator and BRD4 have shown that these proteins are critical for gene expression [69]. In particular, Mediator and BRD4 co-localise at very high levels at “super-enhancers” (SEs): long stretches of sequences with gene regulatory activity that generally drive greater levels of gene expression than regular enhancers [69–72]. We will return to Mediator and BRD4 later in the review to discuss their possible roles in enhancer–promoter communication.

The majority of active enhancers are themselves transcribed, producing enhancer RNAs (eRNAs) in a bi-directional manner [73–77]. These eRNAs are rapidly degraded and might be merely by-products of RNAP recruitment to enhancers [78]. However, studies that knock down selected eRNA have found reduced expression of the corresponding gene targets, suggesting functionality [79–81]. The exact functions of eRNAs are as yet unclear but could include, among others, maintaining enhancer chromatin accessibility [82, 83] and interacting with TFs or cofactors such as BRD4 [84, 85] (reviewed in [86]).

The chain of molecular events following the binding of a pioneer TF to its target sequence (in this case, a promoter-proximal region) has been elegantly documented in a fine-grained time-course experiment following oestrogen receptor activation in a cancer cell line [87]. This study detected multiple “waves” of a progressive recruitment of at least 46 sequence-specific TFs and cofactors, including chromatin remodelling complexes and histone acetyl-transferases, eventually resulting in a derepression of an oestrogen-response gene *pS2* [87].

### Flexibility of enhancer organisation

Given that multiple TF binding sites are the essential building blocks of enhancers, the key question is whether their positioning within enhancers needs to follow any kind of “grammar”, with respect to their particular order and orientation, for an enhancer to function properly (Fig. 1B**)**. The various proposed models for enhancer grammar range from very rigid to flexible, largely dependent on the species, cell types and loci in which they were formulated (reviewed in [29]). At the rigid end, the “enhanceosome” model suggests that the TF binding sites must remain in the same order and orientation for the enhancer to work. This model was based on careful dissection of the virus-responsive interferon beta enhancer, which has eight TF binding sites [88]. The model dictates a set organisation of motifs and spacers consistent with the formation of a heteromultimer of cooperatively bound TFs, each of which directly binds the DNA. Virtually every nucleotide of the interferon beta enhancer is evolutionarily conserved; this is likely because the eight TFs cooperate to form a composite surface that recognises the entire sequence of the enhancer as one effective binding site [88, 89]. In contrast, later studies of *Drosophila* developmental enhancers found evidence for much less rigid motif organisation [90, 91]. At the most flexible end, the loosely organised “billboard” model developed in *Drosophila* implies no cooperativity between TFs for DNA binding and, therefore, the location and spacing between binding sites is not crucial for conserved enhancer function [90, 92]. Enhancers consistent with the billboard model are likely also widespread in vertebrate genomes [55, 93]. An intermediate model for enhancer grammar known as the “TF collective” proposes that a set of the same TFs binds to multiple enhancers but an individual enhancer does not necessarily have the full set of TF binding sites [30, 91]. TF recruitment is therefore facilitated by a combination of DNA binding affinity and protein–protein interactions between TFs. The TF collective model was originally formulated in *Drosophila*, where a set of five TFs bound multitudes of enhancers required for heart development that showed no detectable similarity in their sequence organisation, beyond the presence of binding motifs for some of these TFs [91]. Later studies provided further examples of “TF collective” enhancers. For example, dissection of an enhancer controlling the specification of *Drosophila* leg precursor cells showed that it was robust against motif disruption, but relied on DNA–protein and protein–protein interactions between a particular set of TFs [94]. Similarly, TFs governing serotonergic neuron differentiation showed synergy despite flexible sequence organisation of their cognate enhancers [95]. Flexibility of enhancer organisation with respect to binding site order and orientation was also observed in a massively parallel synthetic enhancer experiment [55]. Biophysical modelling and machine learning approaches have provided more evidence for flexible enhancer grammar [96–98]. For example, modelling the effects of targeting engineered transcription activators and repressors to enhancers suggested that TFs can contribute additively to enhancer function, regardless of their identity [96]. Additionally, a deep learning algorithm applied to binding of pluripotency TFs at base resolution in mouse embryonic stem cells (ESCs) suggested a “soft motif syntax” whereby TFs help each other to bind directly to the enhancer with a lenient distance-dependency between binding sites [98].

The prevalence of flexible enhancer organisation is also supported by rapid evolutionary turnover of enhancers, which exceeds that of proximal promoter regions [29, 99, 100]. In terms of their sequence, there are no known enhancers that are completely conserved at the base level across the animal kingdom [101]. Instead, enhancer conservation is typically “modular”, commonly preserving the sets of binding sites for the required TFs, but often not their exact sequence and orientation [101–109]. Consistent with this, homologous enhancers in a pair of distantly related species can retain their function in the non-host organism despite differences in DNA sequence [101, 110, 111]. Furthermore, even enhancers with extremely high evolutionary conservation may not require the exact preservation of their sequence for their correct function, as was recently shown using CRISPR-mediated mutagenesis [112].

While it is clear that enhancers typically favour the more flexible models of sequence organisation, the degree of necessary cooperation between TFs is less obvious, particularly since TF binding to DNA appears to be very transient [113–117]. Recent technological advances such as single molecule footprinting (SMF) have begun to unpick how TFs work with each other at enhancers [118, 119]. First developed in *Drosophila*, SMF can detect the binding of multiple TFs on a single DNA molecule [116]. SMF showed high TF cooperativity at active enhancers and identified cases where TFs bind independently, sequentially or simultaneously [118]. SMF has also recently been adapted to mammalian cells, detecting widespread co-occupancy of cooperative TFs at enhancers [119]. Notably, at dimeric motif sites, TFs tended to bind initially as monomers with subsequent dimerisation presumably stabilising the DNA–protein complex [119]. Interestingly, at non-dimeric sites, physical interactions between co-bound TFs were not necessary [119], consistent with evidence from *Drosophila* where co-occupied DNA-bound TFs were often spaced quite widely apart (> 50 bp) [118]. Jointly, these results point to synergistic mechanisms between different TFs that do not require direct protein–protein interaction.

How does the flexibility in enhancer organisation result in the robust control of gene expression? In part, this is achieved through tight regulation of the expression of the TFs themselves. One of the best-characterised examples of this is the development of gene expression “stripes” in *Drosophila* embryonic development, which is driven by the expression of pair-rule genes such as *even-skipped* (*eve)* [120]. The stripe 2 enhancer controls *eve* expression in early embryogenesis via binding of tissue-specific TFs [121–123]. This enhancer contains clusters of TF binding sites, with the sites for transcriptional repressors overlapping those for the activators. Activating TFs Bicoid and Hunchback are expressed only in the anterior section of the embryo, while the repressors Giant and Kruppel are expressed in anterior and posterior regions on either side of stripe 2, such that the stripe 2 enhancer is only activated within a 2–3 cell-wide section of the embryo [108, 121, 124]. Another means by which flexible enhancer grammar ensures robust gene expression control is through “suboptimal” TF binding motifs, which reinforce the requirement for TF cooperativity and create opportunities for fine-tuning enhancer activity levels [125–128]. Consistent with this, “strengthening” TF binding sites at developmental enhancers results in aberrant gene expression patterns [125]. Finally, gene control is maintained by the multiplicity of enhancer inputs to the same gene and its regulation by the broader chromatin context, as will be reviewed below.

### The chromatin states of enhancers

Active enhancers reside in open chromatin and occupy regions away from gene TSSs. However, open chromatin regions are not limited to active enhancers, and also harbour other regions including active gene promoters, regulatory elements in a ‘poised’ chromatin configuration (discussed below), as well as some structural regions such as tissue-invariant binding sites for the architectural protein CCCTC-binding factor (CTCF) [129–132]. Post-translational modifications of histone tails provide a more specific readout of enhancer activity. Currently mono-methylation of histone H3 lysine 4 (H3K4me1), associated with the binding of Trithorax/MLL complexes, is taken to be the hallmark of both ‘primed’ and active enhancers, whilst active enhancers additionally tend to associate with the acetylation of histone H3 lysine 27 (H3K27ac) [133–137]. In contrast, the trimethylation of histone H3 lysine 4 (H3K4me3) is typically considered a promoter-associated mark and, in regions proximal to a known TSS, has been used to distinguish the enhancer from a promoter [131]. In addition to the ‘primed’ and active states, enhancers can also reside in a ‘poised’ state exhibiting both H3K4me1 and H3 lysine 27 trimethylation (H3K27me3) that is associated with Polycomb repressive complexes [131, 135, 136, 138]. Finally, some enhancers are marked with H3K27me3 alone and are considered to be “Polycomb-repressed” [139–141]. Many developmental enhancers are found in the poised or Polycomb-repressed state in early development [139, 142, 143], and the functional role of these states remains an area of active research. One possibility is that poised enhancers may be ‘held in check’ for rapid activation [144]. However, enhancer association with Polycomb repressors may also merely serve to suppress enhancer activity [145] or potentially convert these regions to active ‘silencers’ [139].

### Enhancer detection in high throughput

The epigenetic hallmarks of enhancers, including histone modifications and the binding of cofactors such as P300, have enabled their detection at a global scale. Following pioneering studies in a small number of cell types [146–148], large-scale consortia such as ENCODE, Roadmap Epigenomics and BLUEPRINT [131, 149–151] have profiled the epigenetic hallmarks of DNA regulatory elements across cell types in human and mouse cells, as well as in multiple *Drosophila* and *Caenorhabditis* species (through modENCODE) [152, 153]. While the initial efforts focused on cell lines, the analyses have eventually been expanded to multitudes of primary samples, including blood cells and solid tissues. The most recent (phase 3) release of the ENCODE project has compiled an updated registry of predicted *cis* regulatory elements in more than 500 cell or tissue types in human and mouse [131]. Finally, the EpiMap project has combined direct epigenetic mapping with imputation to generate a compendium of 833 reference epigenomes across 18 epigenetic assays [154]. The recently developed CUT&RUN [155] and CUT&TAG [156] methods that require fewer cells and less sequencing compared with ChIP are enabling enhancer identification in an ever-expanding array of cell types and tissues, as well as in single cells [157, 158]. Complementary to this, bespoke methods have been developed to partition the genome into distinct regulatory ‘states’ by integrating information across multiple histone marks accounting for spatial dependency [134, 159–161] to provide an easily interpretable and visualisable readout of enhancer activity in a given tissue.

While enhancer detection based on chromatin profiling has become standard, this approach may falsely detect regions without appreciable regulatory activity, as well as miss some functional elements [146, 162]. For example, enhancers devoid of the classic H3K27ac mark have recently been described [163]; consistent with this, it was shown that this mark is not required for enhancer function, at least in mouse ESCs [164]. These limitations can be partially mitigated by high-throughput techniques that obtain a readout of enhancer activity as opposed to the markers of their chromatin state. For example, methods such as Cap Analysis of Gene Expression (CAGE) can be used to detect eRNA transcripts generated from enhancers, including in single cells [74, 165–167]. In addition, massively parallel reporter assays such as MPFD [168] and STARR-seq [169] make it possible to assess huge libraries of enhancer sequences for transcriptional regulatory activity in vitro [168–175]. Finally, the advent of CRISPR-based techniques streamlines the perturbation analysis of enhancers in vivo. Most relevantly, using the fusions of the ‘dead’ (endonuclease-deficient) Cas9 protein (dCas9) with either transcriptional repressors such as KRAB, or activators such as P300 or tandems of herpesvirus VP16 transactivation domain, enables a guide RNA (gRNA)-targeted inhibition (CRISPRi) or activation (CRISPRa) of theoretically any regulatory region in the genome [176–178]. A recent modification of this approach (known as enCRISPRi/a) combines multiple effector proteins with dCas9 to modulate enhancer (rather than promoter) activity more specifically [179]. To enable CRISPR-mediated enhancer targeting in high throughput, populations of cells are transduced with pooled gRNA libraries, followed by single-cell readouts of enhancer or transcriptional activity from scRNA-seq [180, 181], scATAC-seq [182] and flow cytometry-based RNA FISH [183, 184] to obtain tiled maps of functional regulatory regions for multiple genomic loci in several cell types. In addition to targeting chromatin modifiers using dCas9, knockout screens using wild type Cas9 can also be useful for identifying regulatory elements in high throughput [185–187]. Most of these studies are performed in cell lines; however, CRISPR screening in primary cells is starting to gain momentum [188–190]. Genome editing of enhancers has even been conducted in situ, by injecting guide RNA combinations along with Cas9 mRNA into fertilised mouse oocytes [111]. These novel techniques hold promise for the functional identification of enhancers on a global scale in vivo.

### Natural variation at enhancers and its consequences

While much of the evidence for enhancer function was obtained through perturbing these regions artificially, natural genetic variation at enhancers is commonly observed in human populations, consistent with the relatively low sequence constraint of these regions [191, 192]. Much of this variation has apparently little phenotypic effect due to redundancy both within enhancers (such as through multiple homotypic TF binding sites [191, 193]) and across them (such as through multiple enhancers regulating the same gene, as will be discussed below). Nonetheless, enhancer variation is also known to underlie significant pathologies in both model organisms and humans. In *Drosophila*, for example, point mutations in the binding site for the Kruppel TF within an enhancer controlling the abdominal fate specifier *abd-A* results in its misexpression in a thoracic segment, leading to its conversion to an abdominal one [194]. Likewise, mutations in the enhancers controlling the expression of *Drosophila* homolog of *Pax2* affected mechanosensory bristle development [195]. In humans, a point mutation within a downstream enhancer controlling *PAX6* expression causes the congenital eye malformation aniridia [196]. This mutation was found to prevent PAX6 itself from binding to the enhancer [196]. Point mutations in the ZRS enhancer of the sonic hedgehog (*SHH)* gene are associated with several types of congenital limb malformation and skeletal abnormalities [27, 197, 198] (notably this enhancer is located ~ 1 megabases away from *SHH* and within an intron of another gene, as will be further discussed below). Functional analysis of the ZRS enhancer revealed that these mutations either create gain-of-function binding sites for activating TFs (ETS1 and GABPα) [199], or abolish a repressive TF site [200], leading to ectopic *SHH* expression. Other examples of genes mis-regulated by point mutations in enhancers leading to human disease include *TBX5* (congenital heart disease) [201], *PTF1A* (pancreatic agenesis) [202] and *IRF6* (Van der Woude syndrome) [203]. Capitalising on these observations, sequences predicted to affect TF binding have been used to prioritise causal expression quantitative trait locus (eQTL) variants at enhancers [204–207].

The importance of enhancer variation in human pathology has been further highlighted by genome-wide association studies (GWAS), since GWAS-detected genetic variants associated with complex traits and diseases are typically non-coding, non-promoter associated and highly enriched in enhancers [184, 208, 209]. A classic example is in obesity susceptibility, where trait-associated SNPs are located within the intron of the *FTO* gene, but lead to the dis-regulation of the distal *IRX3* gene [210]. In another example, a systemic lupus erythematosus-associated variant (termed TT > A) situated within an enhancer downstream of *TNFAIP3* has been shown to abrogate NF-kB binding, affecting *TNFAIP3* expression [211, 212]. Subsequent genome-wide TF binding analyses in population cohorts have identified multiple loci of differential TF binding (tfQTLs) across individuals that overlap GWAS variants. For example, genetic variants associated with differential binding of PU.1 were found to underpin GWAS variants associated with blood cell count and autoimmune diseases [213–215]. Beyond differential TF binding, population variation in readouts such as chromatin accessibility [216–219], DNA methylation, histone modifications [220–223] and chromatin looping [215, 224, 225] can provide insights into the genetic determinants of enhancer activity and a functional interpretation of disease associations.

Notably, some enhancer variants that appear functionally neutral when tested under normal steady-state conditions may show “cryptic” effects under stress or upon stimulus response [107, 226, 227]. For instance, eQTL analysis in monocytes stimulated with interferon-γ or lipopolysaccharide has revealed thousands of new variants affecting gene expression, which overlapped nearly 250 variants associated with response to infection and susceptibility to renal and immunological disorders [228]. Likewise, variants associated with type 1 diabetes were strongly enriched at promoter-connected active enhancers in activated, compared with resting, CD4+ T cells [229]. Condition-specific eQTLs were also detected in the cardiomyocytes of patients given the chemotherapy drug doxorubicin, with these loci potentially explaining the differential risks of heart failure upon doxorubicin treatment [230]. The emerging tools for QTL analysis in the single-cell setting [231, 232] have the potential to streamline the identification of regulatory genetic variants with transient effects.

### Similarities and differences between enhancers and promoters

Contrary to the initial assumption that enhancers and promoters were biologically distinguishable elements, it is becoming clear that they share many properties (reviewed in [233]). The ENCODE project, for instance, found that an element might be classified as a promoter in the cell type-agnostic setting, but as a proximal enhancer-like element in a specific cell type [131]. Likewise, proximal and distal enhancer-like elements sometimes display elevated promoter-associated H3K4me3 [131, 234, 235]. Both active enhancers and promoters occupy nucleosome-free chromatin, bind RNAP and can be divergently transcribed [73, 236, 237]. In addition, enhancers within genes are sometimes capable of acting as alternative promoters [238]. Finally, evidence from multiple sources including reporter assays [169, 175, 239, 240], CRISPR [240–242], chromatin mapping [243, 244] and population genetics analysis [207, 245] suggests that certain promoters, termed ‘epromoters’, can also function as enhancers (reviewed in [246]). Notably, while many such elements control house-keeping genes *in cis* (as promoters)*,* they may regulate lineage-specific or inducible genes distally (as enhancers) [240, 247]. In addition, whilst genetic variants at epromoters may affect the expression of both the proximal and distal gene, a large proportion are associated with only the distal one [207]. Therefore, the mechanisms by which epromoters control their target genes *in cis* and *in trans* may not be fully equivalent.

Recently, it has been proposed that cis-regulatory elements exist on a spectrum of enhancer/promoter ability [233, 248]. One key feature determining the position of a regulatory element on this spectrum is likely to be the directionality of its transcriptional output. In a *Drosophila* assay, unidirectionally transcribing elements were more likely to drive strong promoter and limited enhancer activity, whilst bi-directionally transcribing elements had strong potential for enhancer activity and could also function as weak promoters [248]. The “grey area” between enhancers and promoters is supported by evolutionary evidence that some enhancers have undergone genetic mutations allowing them to be repurposed into gene promoters in mammals [100]. Promoters are also sometimes repurposed into enhancers, but at a 13-fold lower rate than enhancer-to-promoter [100]. In terms of their sequence properties, enhancers and promoters tend to differ in GC content, with many promoters and only a subset of enhancers containing CpG islands [74, 249]. Therefore, enhancers predisposed to convert into promoters may have a particular GC sequence composition and presence of 5′ splicing regulatory motif patterns [100].

The potential interchangeability of enhancers and promoters is relevant for functionally interpreting promoter–promoter contacts that are abundantly detected in the genome, as will be discussed later in the review.

## Enhancer–promoter communication in three dimensions

Whilst enhancers were classically tested by cloning them upstream of a core promoter, it quickly became clear that in their native context they are often located large distances away from the genes they control. The classic example of this is the *Shh* ZRS enhancer, which is located within the intron of the *Lmbr1* gene approximately 1 megabase distant from *Shh *[27]. In this chapter, we discuss classic models of how enhancers and promoters can communicate over large distances, focusing on the evidence for the currently prevalent model of direct enhancer–promoter looping and factors thought to mediate it. We then move on to review the emerging exceptions from this model, with potential implications for non-looping mechanisms such as liquid–liquid phase separation. We have left the discussions on the dynamics of enhancer–promoter looping in specific biological settings outside the scope of this review. Several recent articles have provided up-to-date perspectives in this regard for various systems, including lymphocyte development [250, 251], heart development and disease [252], and cancer [253].

### Evidence for enhancer–promoter looping

Historically, several conceptual models were proposed for enhancer–promoter communication. These models were initially applied to the mammalian *β-globin* locus, in which the developmental *β-globin* genes are regulated by a powerful tissue-specific enhancer known as a locus control region (LCR) [254–257]. It was unclear how the LCR could faithfully control the expression of the appropriate set of genes in each stage of erythroid cell development (embryonic, foetal and then adult β-type globin genes). In the “looping” model, the chromatin fibre between the LCR and the gene promoter could loop out until the LCR, as a single unit, is brought close to the promoter in 3D space [257, 258]. Looping between the LCR and gene promoters one by one would mean that gene promoters compete for LCR activation. It was unclear whether the amount of cellular energy required for such dynamic looping was achievable, particularly over increasingly large distances [259]. Therefore, in the alternative “linking” model, TFs binding to the enhancer begin a chain of events whereby multiple proteins bind along the intervening chromatin between the enhancer and the promoter, creating a bridge-like complex [259–261]. In a similar vein, the “scanning” model suggests that proteins recruited by the enhancer slide along the chromatin towards the promoter [262]. According to the linking and scanning models, the protein complexes would eventually become impeded by the presence of transcriptional machinery at the appropriate gene promoter, presumably to prevent spurious gene transcription [263, 264]. At the time, each of these proposed models were equally plausible as there were no appropriate experimental techniques to distinguish between them [259].

The development of novel techniques provided evidence that looping mechanisms are in fact widespread, even though the picture that is currently emerging is more complex, as will be discussed later. Two papers published in 2002 used different novel biochemical techniques to demonstrate that the *β-globin* LCR forms chromosomal loops with the active *β-globin* genes in vivo. Peter Fraser’s team used a method called RNA TRAP (“Tagging and Recovery of Associated Proteins”), in which horseradish peroxidase (HRP)-labelled probes bind to mRNA of specific genes and catalyse the deposition of biotin on chromatin proteins in close proximity with the transcript [265]. Loci that interact with the gene of interest can then be detected by confocal microscopy using fluorescently labelled avidin [265]. In parallel, Wouter de Laat’s team adapted the emerging chromosome conformation capture (3C) technology to explore the *β-globin* locus [266], after it was pioneered in yeast [267]. In 3C, chromatin is fixed by formaldehyde crosslinking and then digested into smaller fragments with a restriction enzyme. Fragments held together in 3D by interacting proteins are ligated (using DNA ligase), with the relative concentration of re-ligated pairs reflecting the frequency (and potentially strength) of their 3D contacts in vivo [267]. 3C confirmed that the LCR comes into close contact with active *β-globin* genes, whilst the intervening inactive genes are looped out [266]. Enhancer–promoter looping occurred in erythroid cells, in which the *β-globin* genes are expressed, but not in brain cells, in which they are not [266]. Further 3C experiments over a developmental time course revealed that the LCR interacts with different *β-globin* genes depending on the stage of erythroid cell development [268]. These findings supported a dynamic looping model rather than a linking or scanning model; neither of which predicted physical proximity between the LCR and the distal gene promoters.

3C technology, which probes the interactions between a single “anchor” with multiple “other end” fragments, was soon expanded to increase throughput. For example, 4C (“Circularized Chromosome Conformation Capture” or “Chromosome Conformation Capture-on-Chip”) employs a secondary restriction digest and inverse PCR to profile all loci interacting with the anchor fragment [269, 270]. In 4C experiments, the LCR was found to interact with multiple highly transcribed genes in the corresponding active cell type (foetal liver) but with different, transcriptionally silent genes in an inactive cell type (brain tissue) [269]. Further modifications of the 3C technology included 5C (few anchors vs few other ends, [271]) and, most significantly, Hi-C that uses biotin pulldown to enrich for ligation junctions, and is thereby capable of detecting theoretically all pairwise interactions between chromatin fragments in the nucleus [272]. The newest modification, Micro-C, replaces restriction enzyme digestion with micrococcal nuclease (MNase) treatment, resulting in smaller fragments and thereby increased resolution of the assay [273, 274]. Originally applied in yeast, it was then adapted to mammalian cells, including human ESCs and fibroblasts [275] and mouse ESCs [276]. Herein, we shall generally refer to 3C/Hi-C/Micro-C methods and their modifications as “3C-derived” methods.

The high complexity of libraries generated by 3C-derived methods necessitates very deep sequencing for identification of individual enhancer–promoter loops. This has nevertheless been achieved in a number of cell lines, providing further evidence for looping in transcriptional regulation [277]. Technologies that combine 3C, Hi-C or Micro-C with sequence capture to enrich the libraries for contacts involving (at least on one end) promoters or enhancers prior to sequencing have made this more achievable. Capture Hi-C (CHi-C) enriches Hi-C libraries (generated using biotin pulldown of proximity ligation junctions) [278–281], while Capture-C instead enriches 3C libraries obtained without biotin pulldown [282–284]. Most recently reported Micro-Capture-C (Micro-C coupled with sequence capture) enables the profiling of pairwise contacts involving selected regions of interest, at up to single-base pair resolution [285]. These enrichment methods have detected multitudes of enhancer–promoter loops in many human and mouse cell types [243, 286–291]. Alternatively, methods such as HiChIP [292] and PLAC-seq [293] (based on Hi-C), or ChIA-PET [294] (based on 3C), use immunoprecipitation to enrich for contacts that are bound to a protein of interest. Using these techniques with antibodies to enhancer- and promoter-associated histone modifications or those to RNA polymerase has also revealed large numbers of 3D enhancer–promoter contacts [244, 295–301].

Imaging techniques provide a complementary way to ascertain the spatial relationships in the genome. The ‘classic’ DNA fluorescence in situ hybridisation (FISH) [302] enables measuring the distance between candidate genomic loci at a single-cell level. DNA FISH has been used to characterise enhancer–promoter loops for several developmental genes such as *Shh* and *HoxD* [303, 304]. Unlike standard 3C-derived methods, imaging techniques obtain a direct distance measurement between the loci of interest at a single-cell level. This gives an opportunity to distinguish between interacting loci that either (a) are moderately proximal in the majority of cells (unimodal behaviour) or (b) have great cell-to-cell variability i.e. are very close in some cells and far apart in other cells (bimodal behaviour) [305]. Single-cell Hi-C can also be used to assess the heterogeneity of chromosomal topologies across cells (reviewed in [306]). However, along with other 3C-derived methods, it can only detect contacts between loci in those cells in which they localise at a close enough proximity to be ligated together (the exact such distance is not fully established). In contrast, DNA FISH gives access to the full distribution of inter-locus distances across all analysed cells [307]. Therefore, 3C-derived methods and FISH cannot be considered merely as “mutually validating” techniques, and they may produce genuinely divergent results, which need to be interpreted carefully [307]. At present, standard DNA FISH cannot reliably detect contacts between loci that are located short genomic distances away (dozens of kilobases) from each other, and have a limited throughput that may miss rare but functionally significant genomic contacts. However, advances in super-resolution microscopy are beginning to mitigate these limitations [308, 309]. Finally, high-resolution live imaging has also paved the way for a real-time visualisation of enhancer–promoter dynamics in vivo using genetic tools and CRISPR-mediated targeting to label these loci with fluorophores [310–312].

The recently developed “forced looping” methodologies have made it possible to probe the causal relationship between chromatin looping and gene expression [313, 314]. The CLOuD9 approach is based on the fusions of two dCas9 variants with the plant molecules ABI1 and PYL1, which dimerise in the presence of a plant phytohormone [313]. The two fusion proteins are directed to the chosen genomic anchors via complementary gRNAs, and phytohormone-induced dimerisation then drives the formation of an artificial chromatin loop. Meanwhile, LADL uses dCas9 fusions of plant proteins that form a heterodimer in response to blue light [314]. Forced looping between *β-globin* and its LCR was shown to rescue *β-globin* expression in K562 erythroid cells (which instead aberrantly express *γ-globin*) in a reversible manner [313]. Forced looping between these loci could also be achieved in HEK293 cells, in which they are heterochromatic, but in this case *β-globin* derepression was not achieved [313]. Hence, as expected, looping alone is insufficient for gene activation by enhancers.

### Factors mediating enhancer–promoter looping

**Fig. 2:**
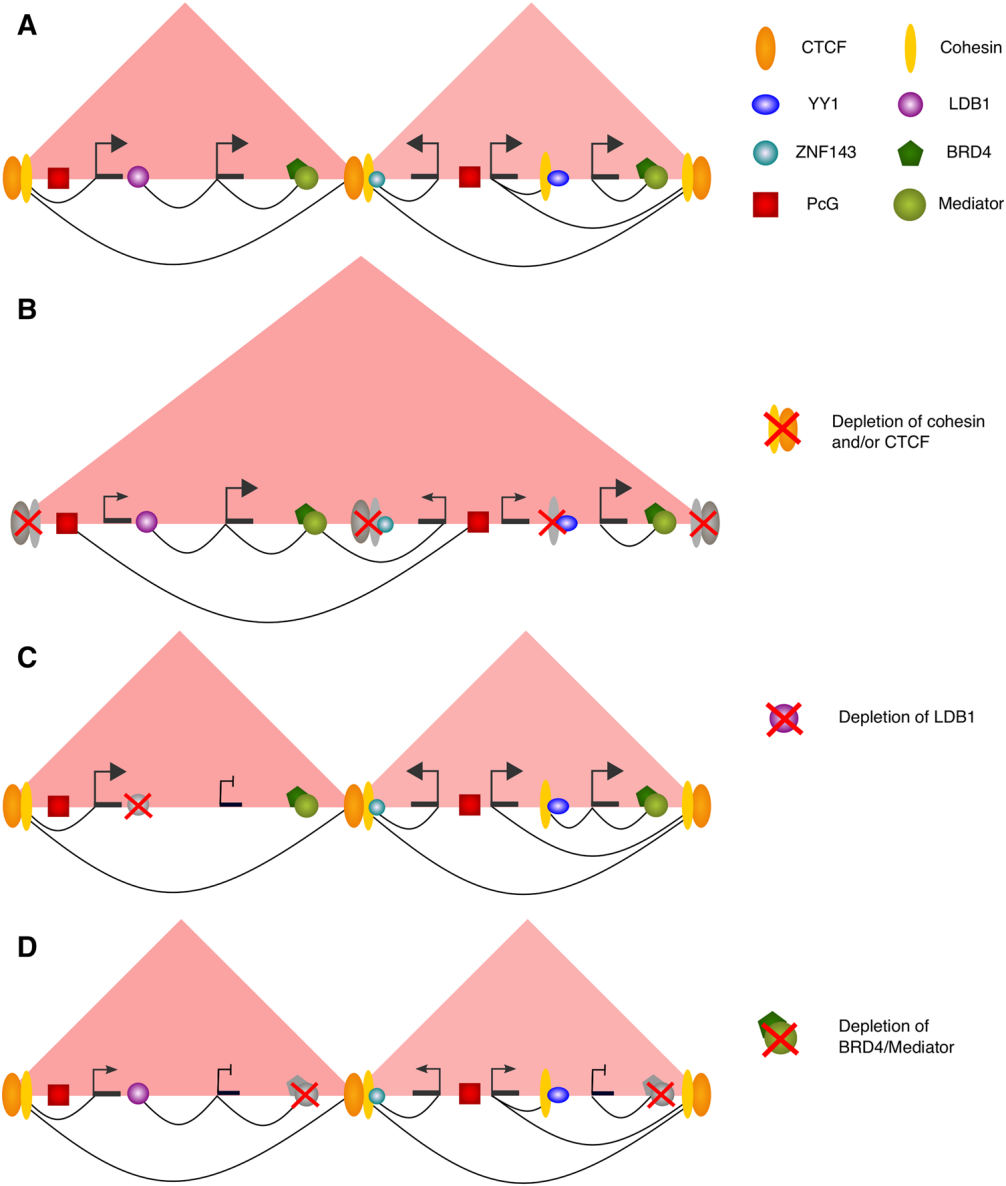
Effect of depletion of selected proteins on enhancer–promoter looping and gene expression. **A** In the wild type, enhancer–promoter contacts (arcs) occur in the context of large-scale contact domains such as TADs (red triangles) facilitated by the joint action of cohesin and CTCF (see “Large-scale chromosomal architecture”). Other cofactors relevant for enhancer–promoter communication (represented in various colours and shapes) can bind independently or in association with CTCF/cohesin at enhancers. Many of these factors are also found at promoters (not shown for simplicity). **B** Removal of CTCF or cohesin abolishes TADs. Long-range enhancer–promoter contacts proximal to TAD boundaries are disrupted, although some short-range contacts remain. In contrast, some long-range Polycomb-associated contacts (arc connecting red squares) and short-range enhancer–promoter contacts appear that spread across the native TAD boundaries. The transcriptional effects of this perturbation, however, remain relatively mild. **C** Removal of LDB1 abolishes an enhancer–promoter loop and leads to decreased gene expression for the affected gene. **D** Removal of BRD4 and/or Mediator does not disrupt enhancer–promoter contacts, but decreases gene expression. See section “Factors mediating enhancer–promoter looping” for details on individual factors and references to primary studies

Following the discovery of chromatin looping in the *β-globin* locus, it was ascertained that several TFs were required for looping between the LCR and the distal genes, including GATA-1, FOG-1, EKLF and NLI/Ldb1 [315–317]. However, whether these factors directly facilitated chromatin looping remained unclear. A direct role in looping in this locus was eventually found for CTCF, whose role we will review in this section, alongside other looping factors (Fig. 2). Some of these proteins, including CTCF, also play a role in organising the global 3D chromosomal architecture, which will be discussed later in the text.

#### CTCF and cohesin

CTCF is a remarkably versatile protein that contains eleven highly conserved zinc fingers with both DNA-binding and protein-binding capacity [318]. Reporter gene assays in selected loci identified context-dependent capabilities of this factor as a transcriptional activator and repressor [319–322]. More unusually, it emerged that CTCF binding has an insulating capacity, effectively blocking signals between enhancers and their target genes in the *β-globin* and *H19/Igf2* loci [323–326]. Dozens of thousands of CTCF binding sites were then identified genome-wide in silico [327] and in vivo [328–330], and it was found that divergent gene pairs separated by CTCF sites had lower than expected correlation of gene expression genome-wide, confirming the insulatory role of CTCF on the global scale [327]. According to the linking or scanning model of enhancer communication, an insulatory role for CTCF could be imagined as a “roadblock” preventing movement of proteins along the chromatin fibre. However, its mechanistic action in the looping model was initially unclear [318]. This was partially reconciled by the finding that CTCF itself can mediate chromatin loop formation at the nucleolar surface [331]. Transgene assays of the human *β-globin* locus then showed that two insulator sites binding CTCF could form a chromatin loop [332]. Indeed, CTCF binding sites flanking the *β-globin* locus were found to physically interact in erythroid progenitor cells, forming a large domain encompassing the LCR and its target genes [268, 333].

A key insight into how CTCF mediates chromosomal looping was the discovery of its association with the cohesin complex [132, 334, 335]. Cohesin had long been known for its role in holding sister chromatids together from DNA replication to chromosomal segregation [336]. The cohesin complex has four core subunits: SMC1, SMC3, SCC1 and SCC3 that join to form a ring-like structure [337–339]. It is currently accepted that this “ring” holds sections of chromatin together, both *in trans* (in sister chromatid cohesion) and *in cis* (when mediating chromomal loops). CTCF is not directly required for cohesin loading and unloading from chromatin, which is mediated by other factors (reviewed in [340]). Instead, it stabilises cohesin loops by blocking the movement of the cohesin ring [341, 342]. Consistent with this, cohesin recruitment to regulatory elements may also occur independently of CTCF, typically in a cell type-specific manner [343].

Beyond mediating specific enhancer–promoter loops, the cooperative action of cohesin and CTCF has emerged as a central organiser of the global chromosomal architecture through a mechanism termed loop extrusion, which will be discussed later in the text (chapter “Determinants of enhancer–promoter selectivity”, section “Large-scale chromosomal architecture”). Surprisingly however, deletion or degradation of cohesin or CTCF in differentiated cells has only mild effects on gene expression [344–349]. Consistent with this, significant numbers of enhancer–promoter contacts remain unaffected upon rapid degradation of these proteins, and some contacts even appear de novo [349, 350]. Mechanisms underlying this phenomenon are still not fully understood, but recent studies suggest that loops that require CTCF might be preferentially long-range (> 100 kb) and situated in loci where enhancers are generally sparse [301, 349]. For example, the contact between versican (*Vcan*) promoter and *Xrcc4* epromoter spanning over 350 kb is significantly reduced, and *Vcan* expression diminished, upon deletion of the CTCF motif at the *Vcan* promoter. Artificial tethering of CTCF to the *Vcan* promoter using a dCas9-CTCF construct effectively rescues the loop and, partially, the expression of *Vcan* [301].

#### Polycomb repressive complexes

Polycomb group (PcG) complexes have long been known to orchestrate the epigenetic silencing of chromatin throughout development. Initially discovered in *Drosophila*, PcG contain two main families: Polycomb Repressive Complex 1 (PRC1), which has E3 ligase activity causing the monoubiquitination of histone H2A lysine 118 (*Drosophila*) or 119 (vertebrates), and Polycomb Repressive Complex 2 (PRC2), which catalyses the di- or tri- methylation of histone H3 lysine 27 [351]. These histone modifications are thought to directly block transcription (reviewed in [352]). In *Drosophila*, PcG complexes aggregate into foci known as Polycomb bodies, which contain co-repressed genes such as *Hox* gene clusters (reviewed in [353]). Within Polycomb bodies, PcG complexes were discovered to mediate chromatin loops between distal loci. For example, 4C and FISH revealed that genes within the *Hox* loci *Antennapedia* and *Bithorax*, which are separated by ~ 10 megabases, are looped together with their associated regulatory elements, ensuring coordinated epigenetic silencing [354]. These findings were corroborated by genome-wide evidence of PcG-mediated looping in embryonic *Drosophila* cells [355, 356]. In mammals, PcG-associated promoters and enhancers also engage in multitudes of looping contacts, particularly in early development [139, 141, 276, 357]. Many of these contacts are undetectable in human naive ESCs where PcG proteins are dispersed throughout chromatin and emerge in primed ESCs [358].

Polycomb-mediated looping is likely to work largely independently of CTCF and cohesin and is potentially even counteracted by the looping activities of these factors in ESCs [357]. Whilst the exact mechanisms of how Polycomb organises the 3D genome are not fully understood, formation of bonds between the PHC1/2 SAM domains of PRC1 complexes was proposed as one potential mechanism [359]. However, knockouts of both PRC1 and PRC2 components affect chromosomal contacts between Polycomb-bound loci [280, 360].

Notably, some contacts between PcG-associated regions, including poised promoters and enhancers in ESCs, are retained after these regions are activated during cell differentiation [141, 143]. Moreover, PRC1 was recently found to mediate chromatin interactions between a subset of active developmental enhancers and promoters in *Drosophila* [361]. Jointly, these results raise the possibility that at least in some cases, Polycomb-mediated chromatin loops may “prime” the connections between regulatory elements in early embryonic cells to ensure their appropriate pairing upon activation later in development.

#### YY1 and ZNF143

YY1 and ZNF143 are both zinc finger TFs with DNA binding domains that have recently been implicated as looping factors mediating interactions between active regulatory elements [295, 362–365]. YY1 was initially proposed to establish loops in the Ig heavy chain locus in pro-B cells [366]. Recent studies have confirmed its global role in connecting cell type-specific active enhancers and promoters in a range of mammalian cell types including mESCs [295] and neural progenitor cells [365]. Meanwhile, ZNF143 binds to promoters globally [367] and was found at the anchors of many lineage-specific enhancer–promoter loops in human and mouse cells [362–364, 368]. Unlike CTCF and cohesin, long-term depletion of YY1 or ZNF143 in mammalian cells causes a significant reduction of enhancer–promoter loops, coupled with substantial changes in gene expression [295, 368, 369] (although short-term YY1 depletion has only a modest effect on both [350]).

The mechanisms by which YY1 or ZNF143 mediate regulatory chromatin contacts are still under investigation. YY1 does not seem to co-localise with CTCF [365] and instead has been proposed to associate with cohesin independently, in a manner analogous to CTCF itself [295]. In contrast, ZNF143 co-localises with both CTCF and cohesin [369], and it has recently been shown that depletion of ZNF143 disrupts CTCF-mediated enhancer–promoter looping [368].

#### LDB1

LIM domain-binding protein 1 (LDB1) is a dimeric cofactor that does not have direct DNA-binding capacity, but interacts with the LIM domain of other TFs to form multi-protein complexes [370]. LDB1 was first identified as a looping factor in the mouse *β-globin* locus [317, 371]. Later studies identified a role for LDB1 in facilitating intra- and inter-chromosomal interactions upon mouse ESC differentiation to myogenic progenitors [372, 373] and in mouse olfactory sensory neurons [373]. In these two studies, deletion of *LDB1* significantly disrupted the expression of the gene programs underpinning appropriate cell differentiation [372, 373]. Ectopic tethering of LDB1 to chromatin is sufficient for loop formation, increased deposition of the enhancer mark H3K4me1 and transcriptional activation [372, 374]. Jointly, these results implicate LDB1 in connecting lineage-specific enhancers with their distal target genes by chromosomal looping. In contrast to YY1 and ZNF143, LDB1 has been shown to form loops independently of both cohesin and CTCF [371, 375]. Consistent with this, the binding of sequence-specific TF PAX3 associates with either LDB1 or CTCF but not both [372]. Likewise, binding sites for Lhx2, another TF that recruits LDB1, are devoid of CTCF and cohesin binding [373].

#### The mediator complex and BRD4

The multimeric Mediator complex is recruited to active enhancers, where it initiates the assembly of the pre-initiation complex and recruitment of RNAP [376]. Mediator was implicated as a looping factor, potentially in association with CTCF and cohesin [377–379]. However, it was shown recently that rapid removal of chromatin-bound Mediator and RNAP does not significantly affect chromosomal architecture, including enhancer–promoter interactions, in spite of triggering widespread transcriptional changes and cell cycle arrest [380].

Mediator recruitment to active enhancers is facilitated by the bromodomain and extraterminal domain (BET) protein BRD4 [70, 71] that plays crucial roles in pluripotency and cancer [71, 381, 382]. Loss of BRD4 leads to global changes in transcription and widespread chromatin decompaction [383, 384], and BRD4 is enriched at enhancers engaged in cohesin-independent promoter contacts in HeLa cells [349]. Nonetheless, removing BRD4 (and, consequently, Mediator) from chromatin using chemical inhibitors did not perturb the majority of enhancer–promoter contacts, despite major changes in transcription [69].

Thus, despite their importance for transcription, Mediator and BRD4 are likely not essential for looping between regulatory elements. Instead, it was suggested that Mediator can aid transcription by supplying TFs and transcriptional machinery to the promoter by diffusion over a small distance, which would not require a direct “bridging” interaction [380]. Whilst these activities might occur in association with the looping contacts facilitated by cohesin and CTCF [380], Mediator and BRD4 may also facilitate liquid–liquid phase separation of enhancers and their target promoters that does not require direct looping [69, 385], as discussed below.

#### RNA polymerase and transcription

The transcriptional process and associated RNA molecules can also influence DNA architecture. Whilst it is established that transcription helps shape up higher-level genome conformation (reviewed in [386]), there is also evidence that it can either disrupt or stabilise specific enhancer–promoter loops. For example, transcription at stimulus–response genes was shown to disrupt chromatin loops [387, 388], potentially through destabilising cohesin association with the chromatin fibre by RNAP machinery [388]. However, a study in bacteria suggests that a related chromatin “ring” (condensin II complex) recovers quickly from the encounters with the elongating RNAP machinery [389].

There is also evidence suggesting that transcription stabilises enhancer–promoter loops [276, 311]. Live cell imaging experiments using a fluorescent reporter gene in the *eve* locus in *Drosophila* detected a causal association between transcription and the enhanced compaction of the locus, bringing the *eve* enhancers into close proximity with the promoter [311]. Moreover, enhancer–promoter loops dissociated tenfold faster in the absence of transcription than when the reporter gene was being transcribed, suggesting that transcription can stabilise contact between these elements [311]. One possible mechanism by which transcription may stabilise loops is through eRNAs (reviewed in [86]), potentially through their interaction with cohesin [81, 378, 390]. Knockdown of eRNAs resulted in decreased enhancer–promoter proximity in human cells [81, 390]. Other proposed mechanisms implicate the transcriptional machinery itself. Transcription by RNAP was found to support some short-range enhancer–promoter and promoter–promoter interactions in fine-scale Micro-C data [276]. However, Promoter Capture Hi-C-based studies did not observe a significant change in enhancer–promoter contacts upon RNAP inhibition, including in cells rapidly depleted of cohesin or CTCF [349, 380].

In conclusion, while cohesin and CTCF remain the established key organisers of chromosomal loops, there is evidence for loops independent of these proteins and of additional looping factors, including potentially RNAP and/or transcription itself. To what extent this explains cohesin-independent contacts and the small effects of cohesin/CTCF deletion on gene expression still remains unclear, prompting the question of whether mechanisms other than direct looping can mediate enhancer–promoter communication, as discussed below.

### Permissive and instructive enhancer–promoter contacts

A long-standing question in the field has been whether enhancer–promoter contacts form concomitantly with the activation of these regions (“instructive”) or, alternatively, precede it (“permissive”) [391]. There are currently ample examples in support of both phenomena. In *Drosophila*, pronounced changes in enhancer activity during embryonic development seem to occur without prominent changes in their 3D conformation [392, 393]. In mammalian differentiation models, in contrast, extensive remodelling of enhancer–promoter contacts is typically observed consistently with enhancer activation [141, 394–397]. In addition, the patterns of promoter contacts in human primary blood cells recapitulate the hematopoietic lineage tree, likely reflecting their gradual remodelling during lineage specification [243]. One possibility is that the pace of development underlies the observed differences in interaction dynamics between *Drosophila* and mammalian models. Nonetheless, a significant minority of enhancers in human ESCs also show pre-existing “permissive” promoter contacts prior to activation [141, 394]. In addition, permissive contacts were also observed in mammalian cells upon TNF-induced enhancer activation [398].

The presence of both instructive and permissive contacts reflects the likely diversity of mechanisms facilitating enhancer–promoter connectivity. One proposed mechanism linking enhancer activity with connectivity is based on the observation that cohesins can be recruited to H3K4me1-marked chromatin, which is associated with the “primed” state of enhancers and the binding of MLL/Trithorax transcriptional coactivators [399]. Additionally, depletion of H3K27ac was shown to result in weaker enhancer–promoter looping in human lymphoma cells [400]. However, the exact determinants of enhancer–promoter loop formation at a given point of time, either prior to or upon the activation of these elements, still remain incompletely understood.

### Enhancer–promoter communication beyond direct looping

Whilst there is now a definitive bulk of evidence in support of physical looping between enhancers and promoters, several recently described phenomena suggest that looping is not the only mechanism of how these regions can communicate, as will be discussed in this section.

#### Deviations from the looping model

The *Sox2* gene has an essential enhancer, the Sox2 Control Region (SCR), which was found by 3C-derived techniques to loop to the *Sox2* promoter (for example, [379, 401])*.* However, live cell imaging over a ~ 25 min interval could not detect close proximity between the SLC and the *Sox2* promoter, even during the bursts of *Sox2* transcription [402]. Moreover, there was no correlation between *Sox2* transcriptional level and enhancer–promoter distance, or any obvious reduction in distance prior to transcription initiation, suggesting that this enhancer may activate *Sox2* transcription via a looping-independent mechanism [402]. While this discrepancy between 3C-derived and imaging-based results is puzzling, the findings are not necessarily contradictory, and may be jointly consistent with either the presence or lack of direct looping. For example, the proximity signal between *Sox2* promoter and enhancer in 3C/Hi-C might be detected if these regions become connected by protein bridges without getting physically closer to each other [403], as would be expected under the linking or scanning models. In addition, 3C-derived methods can pick up rare events that imaging is underpowered to identify [404]. Finally, recent biophysical modelling suggests that functionally causal enhancer–promoter contacts do not have to be temporally concomitant with transcriptional bursts [405, 406]. The exact mechanisms underlying the observed phenomenon, however, remain to be elucidated.

In contrast, a proximal enhancer of the *Shh* gene moves further away from the *Shh* promoter upon activation, according to both 3C-derived methods and imaging [407]. Recruiting activators such as VP64, Mediator and SIX3 to this enhancer also increased the enhancer–promoter distance whilst upregulating *Shh* expression [407]. It has been argued that this might be due to increased enhancer mobility [408] whereby non-thermal energy produced during transcription can “stir” the chromatin [312]. However, whilst this stirring model predicts that the enhancer explores a larger nuclear space, it also moves faster, which would potentially increase the frequency of contacts with the gene promoter [312]. Notably, synthetic activation of the *Shh* promoter, bypassing the need for enhancer activation and causing increased transcription, did not lead to increased distance [407]. Combined, these results would argue against the classical looping model for this enhancer. Given that the looping between the distal ZRS enhancer and *Shh* promoter is well-established, this suggests that multiple enhancers may control the expression of their shared target gene through a combination of looping and non-looping mechanisms.

Finally, multiple lines of recent evidence suggest that many functional enhancers likely do not come into direct contact with their target promoters upon *Drosophila* development (although some “permissive” enhancer–promoter loops are detectable as discussed above) [392]. For example, neither Hi-C nor Micro-C detected a widespread enrichment for enhancer–promoter interactions in early *Drosophila* embryos irrespective of the activity of these elements [393]. In addition, using high-resolution live imaging, it was observed that genes regulated by a single enhancer can localise surprisingly large distances away from each other in 3D (at least 100–200 nm), and yet show coordinated bursts in transcription, which is inconsistent with their direct looping to the shared enhancer [409].

#### Liquid–liquid phase separation

It has been proposed that chromatin can compartmentalise into membrane-less biomolecular condensates in the phenomenon known as liquid–liquid phase separation (LLPS) [410]. Several recent studies argue the case for LLPS as a key mechanism underlying transcriptional control by enhancers [385, 411, 412]. Mechanistically, LLPS occurs when a particular protein increases in quantity to a critically high point, whereupon it spontaneously separates into two phases that contain either a high or a low concentration of the molecule [413]. LLPS is thought to be mediated by weak protein–protein interactions involving intrinsically disordered regions (IDRs) of the protein [412, 414]. Histone proteins themselves might have the ability to form LLPS via intrinsically disordered nucleosome tails [415–417]. In addition, many sequence-specific TFs also have intrinsically disordered low complexity domains within their activation domains that are capable of forming LLPS [412]. In line with this, DNA-bound TFs may have the ability to drive condensate formation via weak, multivalent interactions with recruited cofactors, such as Mediator and BRD4 [69, 385, 414]. The number and density of TF binding sites within enhancers potentially facilitates this by crowding DNA-bound TFs beyond the required threshold required for LLPS formation [418, 410].

The engagement of cofactors Mediator and BRD4 in LLPS could explain why their depletion results in transcriptional changes without significantly affecting chromatin loops [69, 380]. However, looping and LLPS may not be fully orthogonal phenomena, as it was recently proposed that cohesin may induce LLPS of chromatin through forming protein bridges [419]. Likewise, a recent biophysical model could explain the absence of contacts between the SCR and the *Sox2* promoter observed in live imaging data by a combination of looping and phase separation [420].

Whilst LLPS in general has been demonstrated both in vitro and in vivo [421, 422], evidence for chromatin LLPS and its functional significance in gene control is still incomplete. For instance, whilst TFs were shown to form high-concentration stochastic hubs with chromatin in living human cells, overexpression of their low complexity domains beyond endogenous levels was required for LLPS to occur [412]. Furthermore, since high-concentration hubs were capable of transactivation and recruitment of RNAP even without LLPS, the question remains if LLPS is necessary for any genomic locus [412]. It is possible, for example, that LLPS is a distinctive feature of super-enhancers and/or is a consequence, rather than cause, of enhancer–promoter association [411, 412].

The recent interest in LLPS has prompted calls for more rigorous experimental guidelines on its identification and characterisation in the cellular environment [423, 424]. For example, commonly used in vivo approaches like fast fluorescence recovery after photobleaching (FRAP) and sensitivity to treatment with 1,6-hexanediol are not always indicative of LLPS [423]. Nonetheless, it is compelling to consider if LLPS could be a mechanism of enhancer–promoter communication that is complementary to chromosomal looping. First, LLPS could underlie gene control by “contactless” enhancers, uniting them with their target promoters and supplying them with the required proteins without the need for physical proximity [52]. LLPS could also provide a mechanistic explanation to the classic “transcription factory” model [425], whereby specific activators or repressors are assembled in high concentration at appropriate genomic loci [426, 427]. For example, it has been suggested that Mediator and RNAP molecules may accumulate in condensates at super-enhancers, and these condensates could facilitate efficient loading of the transcriptional machinery to active gene promoters [426].

Notably, phase-separated condensates might form and dissolve in accordance with local chromatin features, such as histone tail acetylation, the presence of linker histone H1 presence and inter-nucleosome spacing, as well as the concentrations of recruited TFs [52, 415]. Molecules such as RNAP might also be passed between different condensates based on their post-translational modifications [426, 428]. Therefore, such “factories” formed by LLPS may be extremely transient [52], consistent with the dynamic and “bursty” nature of transcription. Finally, an important feature of LLPS is that it has been modelled quantitatively, at least in simpler systems, building on the foundations of soft matter physics [429]. These models facilitate formal testing of the involvement of LLPS in chromatin organisation and prediction of its implications in gene control.

## Determinants of enhancer–promoter selectivity

It is clear from both genome-wide profiling and perturbation studies that, of the multitudes of enhancers in a locus, not all of them provide input to every promoter [430, 431]. The underpinning global genomic context, discussed immediately below, has emerged as a key factor determining enhancer–promoter selectivity, providing a means to mitigate and modify the effects of linear genomic distance. However, it still does not explain all observed effects, suggesting the involvement of other phenomena, such as the mutual compatibility of specific promoters and enhancers that is discussed later in this chapter.

### Large-scale chromosomal architecture

#### Products of loop extrusion: TADs and stripe domains

It has become evident from early analyses of Hi-C data that metazoan genomes are organised into megabase-scale segregated domains that are often invariant between cell types, termed topologically associating domains (TADs) [432–434]. While initially, the mechanisms underlying TAD formation and maintenance were unclear, an explanation has eventually been provided by the model of chromosomal loop extrusion [342, 435]. Loop extrusion was initially proposed theoretically based on molecular dynamics simulations [342, 435] and then validated in single-molecule assays [436, 437]. Under this model, cohesin in interphase nuclei continuously extrudes chromosomal loops until it encounters a boundary, which prevents further extrusion. In the classic scenario, this boundary is presented by CTCF binding to its sequence motifs located in a divergent orientation at either end of the loop [277, 438–440], consistent with CTCF’s established role as an insulator-associated protein. Loops stalled by CTCF or other boundaries constrain chromosomal contacts, giving rise to “loop domains” detectable in Hi-C data. Notably, TADs correspond to only one level of a hierarchy of such “loop domains”, with super-/sub-TADs and insulated neighbourhoods potentially corresponding to other levels [434, 441, 442]. It has been suggested, however, that the level of the hierarchy corresponding to TADs is “functionally privileged”, as their boundaries show the highest enrichment in CTCF binding and conservation across cell types [443].

TAD boundaries are classically defined through a change in the predominant direction of chromosomal contacts observed in Hi-C data [444]. The majority of specific enhancer–promoter loops follow this pattern and are highly enriched within, compared with across, TAD boundaries [277, 401, 405]. Consistent with this, disruption of TADs can cause promiscuous enhancer–promoter contacts and ectopic gene expression leading to developmental aberrations and disease (reviewed in [445]). For example, loss of TAD insulation between limb-specific enhancers associated with *EPHA4* expression and a set of adjacent genes, including *WNT6* and *PAX3,* results in their misexpression and abnormal limb development in both mice and humans [446]. More recently, TAD fusion was also proposed as a mechanism leading to several disease phenotypes upon genomic duplications in the *SOX9* locus [447]. However, TAD structure in general may play less definitive a role in gene regulation than originally thought. Most strikingly, rapid depletion of cohesin or CTCF quickly abolishes TADs but, as mentioned above, has only relatively mild effects on gene expression [301, 345–347, 448]. Consistent with this, the considerable TAD re-arrangements observed in *Drosophila* balancer chromosomes compared with their wild-type counterparts result in changes in the expression of only ~ 10% of genes [449]. Likewise, targeted deletions of TAD boundary-forming CTCF sites in the *Shh* locus did not entirely disrupt the developmental regulation of *Shh* expression [450, 451].

The less pronounced than expected role of TADs in gene control can be partially explained by the ability of some enhancer–promoter contacts to form and/or be maintained independently of TAD constraints. For example, Capture Hi-C analyses in multiple cell types consistently detect enhancer–promoter contacts that cross TAD boundaries [141, 243]. Recent mechanistic experiments suggest that the insulating effect of TAD boundaries may be inversely dependent on enhancer strength [405]. In addition, single-cell studies show both a substantial cell-to-cell heterogeneity of TADs and large numbers of inter-TAD chromosomal contacts [448, 452]. Furthermore, in the developing *Drosophila* embryo, enhancer–promoter loops are formed before TADs, and likely also precede the onset of zygotic transcription [453, 454]. *Cis-*regulatory contacts were also shown to emerge prior to the formation of TADs in murine cells exiting mitosis [455].

Another layer of complexity in the relationship between chromosomal domains and specific enhancer–promoter contacts arises from the fact that active promoters and enhancers can likely give rise to TAD boundaries even in the absence of convergent CTCF sites [401]. Disentangling the cause-and-effect relationship is particularly challenging in the case of a flavour of TAD-like structures known as stripe domains (from the way they appear on Hi-C maps) [276, 456, 457]. Stripe domains tend to anchor at a single CTCF binding site (as opposed to two divergent CTCF sites in the classic case for TADs), which points towards a nearby site for the cohesin-loading factor NIPBL [456, 457]. It has been suggested that they arise from one-sided loop extrusion, facilitated by the loading of cohesin to lineage-specific enhancers [457]. However, experiments using finer-scale mapping with Micro-C have revealed even smaller, nested stripes that may occur independently of loop extrusion [276]. These 10–50 kb pair stripes are enriched in promoter–promoter and enhancer–promoter interactions and are largely unassociated with CTCF/cohesin binding [276].

In conclusion, whilst there is clear evidence for a role of some TADs in gene control, the emerging view is more nuanced than originally envisaged. Rather than being fully insulated structures that predetermine enhancer–promoter contacts, TADs and related loop domains can be seen as flexible hierarchical features of genomic organisation that loosely contain, but also can potentially be generated by, enhancer–promoter contacts.

#### A/B compartments and LADs

Beyond TADs, other aspects of the global chromatin structure may also play a role in enhancer–promoter communication. Most prominently, contacts from active chromatin and heterochromatin regions appear largely segregated into separate compartments (referred to as A and B, respectively) [272]. These “A/B” compartments are dynamic across cell types and conditions. Most strikingly, around 1/6^th^ of the genome undergoes compartment changes during the 24 h circadian cycle in mouse adult liver [458]. The A/B compartment structure does not disappear (and is in fact even strengthened) upon cohesin/CTCF depletion [344, 346–348], but, like TADs, dissolves in mitosis [459]. It has been suggested that A/B compartments may be facilitated by affinity of heterochromatin regions to each other [460, 461]. Whether contacts between active *cis*-regulatory elements (particularly those independent of cohesin/CTCF) also participate in compartment formation currently remains an open question.

A subset of B compartments corresponds to lamina-associated domains (LADs) [462]. LADs represent ~ 500 kb-long chromatin regions localised at the nuclear periphery. These regions are generally gene-poor regions with low transcriptional activity. Mechanisms by which LADs promote transcriptional repression are not fully understood, but may include sequestration of genes and their regulatory elements in heterochromatin foci [462]. Whilst many LADs remain generally invariant across cell types and conditions, others can move away from the nuclear periphery, for example, upon T cell differentiation [463]. This relocation is associated with the activation of the respective regulatory elements and their increased proximity [463]. Recently, some A compartment regions consisting of H3K9me2-marked chromatin were also found to localise at the nuclear periphery [464]. These domains (dubbed H3K9me2-Only Domains; KODs) are distinct from LADs and have been suggested to represent an “intermediate” chromatin state with a role in regulating tissue-specific gene expression [464].

### Genomic distance

Chromosomal contacts follow the law of constrained Brownian motion (reviewed in [465]), with the contact probabilities showing a strong power–law dependence on the “linear” genomic distance [272, 466]. In mitosis, where TADs and compartments dissolve, genomic distance becomes the predominant factor governing the frequency of chromosomal contacts across the genome [459]. However, even in interphase nuclei, genomic distance continues to be a reasonable predictor of functional enhancer–promoter contacts, particularly in the short to medium range (< 100 kb), as evidenced by high-throughput enhancer perturbation data [183, 184]. Recently, Zuin et al. investigated the positional effects of enhancer–promoter contacts by embedding an enhancer into a PiggyBac transposon and integrating it ectopically next to a reporter gene [405]. PiggyBac “jumps” resulted in the enhancer relocating away from the promoter over a range of distances, but mostly within the same 560 kb TAD as the reporter gene, enabling a comparative analysis between different clones. In this systematic study, the probabilities of enhancer–promoter contacts showed a strong dependence on linear distance within the TAD and fell sharply near and across the TAD boundaries [405].

The effects of distance, however, are likely not absolute, even within the same TAD. For example, analysis in the *Shh* locus suggests that within-TAD chromosomal contacts there do not show a pronounced distance dependence [467]. In addition, many computational algorithms for the analysis of data from Hi-C and related techniques explicitly correct for distance decay, identifying “significant interactions” based on how unusual their contact frequency is at a given distance [277, 468–471]. Promoter-interacting regions identified using these approaches typically show enrichment for enhancer-associated chromatin marks and disease-associated variants, and in a number of cases their target genes have been validated directly (reviewed in [431]). In contrast, the model of Zuin et al. [405] discussed above was based on the potentially “contactless” SCR and its target *Sox2* promoter. Consistent with this, in the ectopic context this enhancer–promoter pair did not show prominent specific interactions, despite the clearly detectable transcriptional effects of SCR [405]. Therefore, the extent of distance dependence of enhancer–promoter contacts likely depends on the underlying genomic context and potentially also on the properties of specific enhancers and promoters.

### Enhancer–promoter compatibility

Even within the same TAD, enhancers are not always fully promiscuous and can sometimes “skip” nearby genes, in favour of more distal targets [472, 473]. Sequence compatibility between enhancers and promoters has been proposed as one of the factors underlying their pairing [474]. For example, enhancer trap experiments in *Drosophila* that used randomly integrated reporter genes driven by a TATA- and a DPE-containing promoter, respectively, showed that some endogenous enhancers had a preference for either of the two promoters [475]. More recently, a massively parallel reporter assay in *Drosophila* cells revealed distinct groups of enhancers that preferred one of two types of core promoter, derived from either a housekeeping or a developmental gene and enriched for different TF binding motifs [247]. Notably, this preference was consistent with the tissue-invariant or tissue-specific activity of the enhancers themselves [247].

Beyond the *Drosophila* model system, there is currently little evidence for inherent enhancer–promoter compatibility. Further insights on potential selectivity may yet come from machine learning methods that are gaining the ability to predict enhancer–promoter pairs from sequence alone in mammalian cells [476, 477]. While currently these are generally “black box” methods, such as deep neural networks, advances in interpretable machine learning may provide a handle on the specific sequence features of these elements that are predictive of their compatibility. In addition, at least some of the perceived selectivity of enhancer–promoter contacts could be due to other factors, such as enhancer competition for promoters. For example, it was shown that deleting or inactivating the target promoter of a particular enhancer releases the enhancer to activate other local promoters [478]. In addition, biophysical modelling suggests that the same enhancer may have vastly unequal effects on different promoters depending on their intrinsic sensitivity to enhancer–promoter contacts (irrespectively of the properties of a given enhancer) [406].

The findings discussed in this chapter show a diversity of the aspects of nuclear organisation that regulate enhancer–promoter communication. The outstanding question is how exactly these multiple aspects work together to establish appropriate enhancer–promoter relationships. One way to conceptualise this complexity at the current level of understanding is to assume enhancer–promoter pairing to be a largely stepwise process, as proposed recently by Schoenfelder and Fraser [431]. In this view, enhancers are “selected” for activation *in cis* by TFs, following which their contacts with target promoters are then “facilitated” by the underlying chromosomal topology and then further “specified” by parameters of enhancer–promoter compatibility [431]. Further functional analyses and quantitative modelling of enhancer–promoter communication will help to validate and improve our understanding of these phenomena.

## Beyond pairwise enhancer–promoter relationships

It follows from the rather flexible requirements for enhancer–promoter selectivity (discussed above) and the very large number of enhancers active in a given cell at a time that genes often receive output from multiple enhancers. It is also currently clear that the same enhancer can control multiple genes. Here, we discuss the functional and structural considerations around this multiplicity of enhancer–promoter relationships.

### Evidence for multiplicity of enhancer–promoter connections

#### One promoter—many enhancers

Enhancers vastly outnumber promoters in human and mouse genomes [131], and consistently promoters often contact more than one enhancer in the same cell type [141, 243, 278, 279]. For example, PCHi-C showed that active promoters contacted a median of two active enhancers in human primary blood cells [243]. CRISPR-based approaches have confirmed that promoters often receive signals from multiple functional enhancers [479, 480]. In developing mouse limb tissue, for instance, genes were regulated by a median of three functional enhancers and some genes, which coded for limb-specific TFs, had more than ten associated enhancers [480]. Promoters can also engage multiple enhancers in different cell types or at different developmental stages. For example, in the well-characterised mammalian *Hox* gene cluster, activation of genes with crucial roles in development is tightly controlled at each developmental stage by complex enhancer networks [481, 482]. In *Drosophila*, *eve* expression is controlled by five separate enhancers to produce stripe patterning in the embryo [483].

Multiple enhancers associated with a single promoter in a given cell type may be conceptualised as *cis-*regulatory units (CRUs) [141]. Analyses in human ESCs and early ES-derived neural progenitors found that, as expected, enhancers within a CRU tended to have the same activity state in a given cell type [141]. However, this effect was not absolute, with 20% of CRUs detected in these cells being “dual-state”, i.e. containing a mixture of active and poised or Polycomb-repressed enhancers [141]. Upon early neural differentiation, about a third of single-state CRUs in human ESCs became dual-state, while around half of all dual-state CRUs became single-state [141]. Changes in CRU state were predominantly driven by coordinated alterations in the chromatin state of enhancers and their promoter contacts, and were associated with significant changes in gene expression [141]. Notably, the chromatin state of the promoter at dual-state CRUs typically corresponded to the majority state of its enhancers, consistent with a competition of opposing regulatory effects at the promoters.

#### Many promoters—one enhancer

The same enhancer can also regulate more than one target gene—either in different spatiotemporal settings or in the same one. An example of the former is found in the *Drosophila Hox* locus, where one enhancer regulates either *pb* or *zen2* depending on the developmental stage via distinct chromatin loops [484]. Examples of enhancers contacting multiple genes in the same cell type are also abundant in data from Hi-C and related technologies. While these data cannot ascertain whether these contacts occur in the same cell, recent evidence suggests that this may indeed be the case. For example, the LCR in the *β-globin* locus can simultaneously engage two adult globin genes, *Hbb-b1* and *Hbb-b2,* while skipping over embryonic globin genes, in mouse fetal liver cells [485]. Likewise, evidence from live imaging shows that an enhancer located in-between two reporter genes can initiate simultaneous transcriptional bursting from both gene promoters [310].

#### Enhancer chains and networks

In addition to multiple direct enhancer–promoter contacts, there is also evidence for enhancer–enhancer interactions that create an enhancer “chain” or network [486–489]. In this mechanism, not all of the enhancers need to contact the gene promoter directly; rather, the signal could be passed along the chain. Evidence from Hi-C data analysis has suggested that the “first” enhancer in the chain, i.e. the enhancer with direct looping to the gene promoter, is often more distal than the “second” enhancer, attracts more tissue-specific factors and is more enriched for eQTLs than other enhancers within the chain [490]. Consistent with this, recent dissection of an enhancer chain in the *INK4a/ARF* locus revealed that perturbing any promoter-interacting enhancer within the chain abolished the functionality of the entire network, but deleting the enhancers further down the chain had more moderate effects [491]. Furthermore, deletion or inhibition of any one of the promoter-interacting enhancers caused disruption of H3K27ac and eRNA levels on the other enhancers in the network [491]. Combined, these data suggest that the “first” enhancer is responsible for connecting with the promoter and initiating gene transcription, but also relies on signals from further down the enhancer chain.

### Promoter–promoter contacts

3C-derived methods commonly detect multitudes of promoter-promoter contacts, typically in addition to promoter-enhancer contacts of the same genes. In some well-characterised cases, multiple promoters engage in combinations of pairwise interactions with each other, suggestive of promoter-promoter “networks”. These include contacts between the promoters of *Hox* genes in ESCs (in which they are inactive) that were shown to depend on Polycomb Repressive complex 1 [280]. A similar network of contacts is also detectable between the active promoters of multiple histone genes in many mammalian cell types [243, 279, 471, 492]. These two examples may reflect co-regulatory relationships between the interacting promoters, consistent with the classic “transcription factory” model [425]. In addition, recent evidence that promoter-promoter contacts within nested stripe domains preferentially involve transcriptionally active, rather than inactive, promoters [276], as well as the existence of epromoters (discussed above), suggest that some promoter-promoter contacts likely have a direct cis-regulatory role similarly to enhancer–promoter contacts. This concept has also recently been supported by computational analyses based on Hi-C data [493].

### Transient contacts or enhancer hubs?

The subsections immediately above provide an empirical view on the multiplicity of enhancer–promoter and promoter-promoter relationships. How can such configurations be achieved structurally? Whilst 3C-derived data have indicated that interactomes comprising multiple interacting elements may be common in mammalian genomes [360, 488, 494–496], these data alone cannot prove the simultaneous association of multiple loci. This is because in their standard form, 3C-derived methods detect pairwise ligation events. Therefore, whilst these findings might represent multi-way “hubs”, they might equally represent a superposition of multiple independent contacts between enhancers and promoters. In order to explore this question further, several techniques have been developed that circumvent the limitations of standard 3C-derived methods.

Genome Architecture Mapping (GAM) is one technique that enables probing multi-way chromosomal contacts [497]. GAM works by laser microdissection of cryopreserved nuclei, followed by DNA sequencing within a given slice, to find all loci that were nearby in 3D space [497]. Another inherently non-pairwise method is Split Pool Recognition of Interactions by Tag Extension (SPRITE), which uses chromatin crosslinking but no proximity ligation [403]. Instead, barcode signatures are created for restriction fragments that are then sequenced and clustered to identify chromatin complexes [403]. While both methods could successfully reveal multi-way contacts across TADs and chromosomes, their resolution currently limits robust detection of individual CRUs. Multi-way relationships can also be inferred using 3C-derived methods, provided that multiple pairwise ligation events are detectable in the same sequencing read. This was achieved with very short (~ 200 bp) restriction fragments (Tri-C [498, 499]), or by using very long (~2 kb) reads generated with Oxford Nanopore seqeuencing (MC-4C [485]). Both technologies, applied in the *α*- and *β- *globin loci respectively, provided evidence for higher-order hubs, containing three and more regulatory elements [499, 485]. Finally, advances in super-resolution imaging (reviewed in [500]) now enable the visualisation of multiple interacting loci to the resolution of a few kilobases in single cells [448, 501–503]. Hi-M is one such technique that combines visualisation of chromatin interactions and transcription in single cells [501, 504]. Hi-M was recently employed in the developing *Drosophila* embryo to show that *cis*-regulatory elements cooperate to form hubs early in development prior to transcription, facilitated by the pioneer factor Zelda [453]. Notably, in addition to multiple enhancers, hubs detected by these techniques sometimes contain multiple promoters, corroborating the evidence for promoter-promoter contacts from the conventional 3C-derived methods [453, 498].

Evidence from live-imaging studies at candidate loci suggests that physical contacts between enhancers and promoters are often highly dynamic [310, 311, 448]. For example, in the *β-globin* locus, interactions between the LCR and the *γ-globin* or the *β-globin* genes can switch very rapidly on the same DNA strand [505]. Nonetheless, the existence of bona fide hubs could be explained by the phenomenon of LLPS, rather than direct looping, whereby various interacting regulatory elements are integrated into phase condensates [453, 506, 507]. These LLPS-mediated hubs could be formed or dissolved by local changes in factor concentrations or other biochemical properties that still allow for a dynamic, adjustable system for gene expression control.

### Pioneer and maintenance enhancers

So far we have been considering multiple active enhancers in a unit as generally serving the same role. However, there is a possibility that some enhancers may also help others to associate with promoters, possibly in addition to their direct activity on those same promoters. For example, in the vertebrate *Hox* cluster, a strong enhancer situated outside of the gene locus could prime the region, allowing for the emergence of novel enhancers [508], leading to the notion of “pioneer enhancers” [509] in an analogy to pioneer TFs. Consistent with this model, it was recently shown that deletion of a relatively weak distal enhancer in the mouse *Sox9* sex determination locus leads to sex reversal, while the phenotypic effects of deleting stronger and more proximal *Sox9* enhancers are much less pronounced [510]. Analysis of enhancer chains suggests that the promoter-connected enhancer is often more distal than downstream enhancers in the same chain, yet binds more tissue-appropriate TFs, suggesting that enhancer chains might involve pioneer enhancers [490]. One possibility is that pioneer enhancers are required for gene induction, while other enhancers are involved in the maintenance and fine-tuning of gene expression [511, 512].

**Fig. 3:**
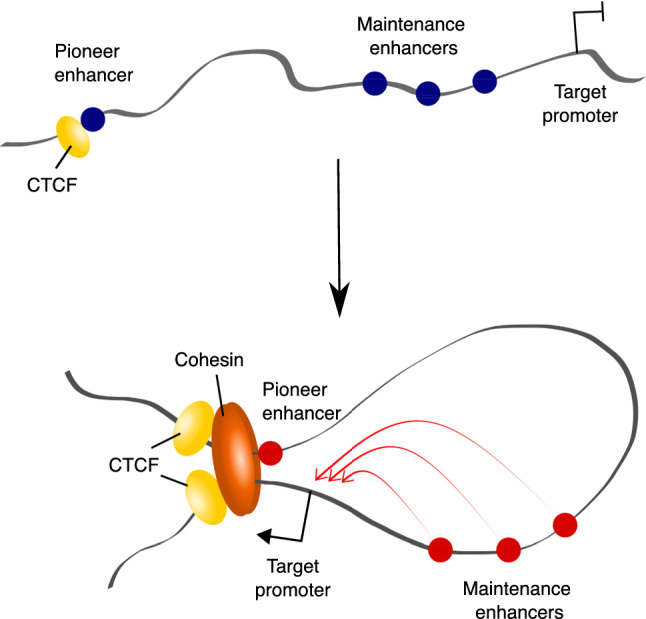
A model of pioneer and maintenance enhancers. A distal pioneer enhancer, potentially bound by CTCF and/or cohesin, initiates a chromatin loop with the gene promoter and increases accessibility of the locus. Subsequently, more proximal cohesin-independent maintenance enhancers become activated and initiate gene transcription. Blue and red circles denote inactive and active enhancers, respectively

Theoretically, cohesin/CTCF-associated enhancers are good candidates for pioneer enhancers for a number of reasons, most important of which is the selective requirement of cohesin for inducible gene control [513]. Cohesin-dependent enhancers also tend to be longer-range than their cohesin-independent counterparts [301, 349, 514]. One possibility therefore is that looping orchestrated by cohesin/CTCF-bound pioneer enhancers enables cohesin-independent contacts [349] (Fig. 3), which may be mediated by other factors (such as, for example, LDB1) and mechanisms, including LLPS. Further mechanistic analyses of multi-enhancer control of inducible genes will be needed to validate this model directly.

## Transcriptional effects of enhancer action

What are the consequences of enhancer action on promoters? Conceptually, the simplest model would suggest that when an enhancer comes into proximity with a compatible promoter, it transmits to it a “dose of activation” that results in a certain level of transcriptional output [107]. (In view of the evidence reviewed in the previous sections, we can assume that this proximity can be achieved by either direct looping or otherwise, and may be potentially facilitated by a pioneer enhancer). The “dose of activation” model, although necessarily crude, fits well with the current understanding of the generally transient contacts between regulatory elements, TFs and cofactors, as well as with the flexibility of enhancer organisation discussed above. This model is also supported by the fact that transcription itself is discontinuous, occurring in “bursts” during which RNAP complexes transcribe one by one from the TSS producing one or more transcripts, followed by a period of transcriptional silence (reviewed in [515]). Evidence from live imaging and single-cell transcriptomics indicates that enhancers increase the frequency of transcriptional bursts without significantly affecting their amplitude and duration [310, 505, 516].

One implication of the “dose of activation” model is that multiple enhancers controlling the same gene are expected to provide largely additive inputs to the promoter. In support of this notion, the number of enhancers correlates with the expression level of a gene across cell types [243, 278, 279, 349]. Likewise, the transcriptional effects of enhancer deletions within extended super-enhancer loci are also consistent with the generally additive effects of individual enhancers [517, 518]. Finally, considerations related to the “dose of activation” view also lie at the foundation of the Activity-by-Contact (ABC) model for estimating the contribution of a given enhancer to a gene’s transcriptional output. In ABC, each enhancer is considered separately and the “dose” of activation it contributes to a promoter depends on its individual activity *in cis* and the frequency of the respective enhancer–promoter contact [183, 184]. The ABC model showed a good performance in predicting the transcriptional effects of CRISPRi-mediated enhancer perturbations (one-by-one) in dozens of loci in human cell lines, particularly for the more proximal enhancers [183, 184].

However, it has long been known that additivity in enhancer action is not absolute, and some enhancers work together either synergistically or sub-additively. For example, a mixture of additive, synergistic and sub-additive effects was demonstrated in a live imaging analysis of candidate developmental enhancers in *Drosophila* embryos [519]. Sub-additivity may also underlie some cases of functional enhancer redundancy, such as for *Drosophila* “shadow enhancers” that can “back up” important developmental enhancers and enable them to accumulate deleterious mutations [520–522]. In turn, synergistic effects were observed in mammalian cell fate determination systems, for example, at *Fgf5* enhancers during the differentiation of mouse embryonic stem cells to epiblast-like cells (EpiLCs) [523] and at up to 20% enhancers upon transdifferentiation of human leukemia B-cells into macrophages [524].

Some deviations from enhancer additivity could potentially be explained by steric constraints arising from enhancer competition [519] or involvement of “pioneer enhancers” [525], but these considerations may not readily explain other reported cases [523, 526]. Recent insights into the biophysics of enhancer–promoter relationships expand the scope for possible scenarios underpinning these effects. Most importantly, dynamic modelling studies have challenged the direct relationship between enhancer–promoter contacts and transcriptional bursting. Instead, they propose that an enhancer’s contact with a promoter may facilitate the transition of the promoter from a low-activity state to a highly active state [405, 406], which in turn may generate bursts at a higher frequency. Mechanistically, this could be achieved if rather than directly initiating transcriptional bursts, the “doses of activation” provided by enhancers are accumulated at promoters through some intermediate molecular tags (such as histone modifications, transcription factors or RNAP itself), with a certain level of such “tags” required for transition to an active state [406]. This way, promoters may have a “memory” (hysteresis) of enhancer action [405, 406], reviving some early debates on this topic [527]. It also follows from these models that the frequency of enhancer–promoter contacts may have non-linear effects on transcriptional output, consistent with experimental observations [405, 503].

In theory, the functional “decoupling” of enhancer–promoter contacts from transcriptional bursting may accommodate the existence of multi-enhancer hubs and non-looping mechanisms of enhancer–promoter communication. For example, such hubs formed through phase separation may help to cluster RNAP at gene promoters, prior to RNAP pause release that directly results in a transcriptional burst [506, 528, 529]. However, the exact interplay between these phenomena, and the extent to which they are jointly capable of explaining the complexity of gene control by multiple enhancers, remains to be fully understood.

## Concluding remarks

Research over the last few decades has provided ample evidence for the key role of enhancers in metazoan gene regulation and the importance of their aberrations in disease. We now also have efficient tools for detection of enhancers and their target genes genome-wide in cell populations and, increasingly, in single cells. Jointly, these advances have improved our understanding of the regulatory logic and molecular mechanisms underpinning enhancer activity *in cis* and *in trans*. However, the bulk of evidence of enhancer–promoter communication to date is based on 3C-derived methods that presume physical looping between these elements and necessitate a largely pairwise view on their relationships. Modern technologies gradually make it possible to transcend these limitations, enabling more direct investigations of non-looping mechanisms and multi-enhancer regulatory logic. We are also beginning to expand our conceptual understanding of enhancer types and states beyond a simple “on” and “off”, with emerging notions such as poised and pioneer enhancers. Single cell methods, particularly those based on high-resolution live imaging, combined with powerful genetic and epigenetic perturbation tools and state-of-the-art computational analysis paradigms, have the potential to significantly advance our ability to probe these concepts, paving the way towards comprehensive quantitative models of *cis*-regulatory gene control.
